# Hyperforin Potentiates Antidepressant-Like Activity of Lanicemine in Mice

**DOI:** 10.3389/fnmol.2018.00456

**Published:** 2018-12-12

**Authors:** Bartłomiej Pochwat, Bernadeta Szewczyk, Katarzyna Kotarska, Anna Rafało-Ulińska, Marcin Siwiec, Joanna E. Sowa, Krzysztof Tokarski, Agata Siwek, Alexandre Bouron, Kristina Friedland, Gabriel Nowak

**Affiliations:** ^1^Laboratory of Neurobiology of Trace Elements, Department of Neurobiology, Institute of Pharmacology, Polish Academy of Sciences, Krakow, Poland; ^2^Department of Physiology, Institute of Pharmacology, Polish Academy of Sciences, Krakow, Poland; ^3^Department of Pharmacobiology, Faculty of Pharmacy, Jagiellonian University Medical College, Krakow, Poland; ^4^Université Grenoble Alpes, CNRS, CEA, BIG-LCBM, Grenoble, France; ^5^Pharmacology and Toxicology, Institute of Pharmacy and Biochemistry, Johannes Gutenberg University Mainz, Mainz, Germany

**Keywords:** depression, NMDA - receptor, hyperforin, lanicemine, TRPC 6, ketamine

## Abstract

N-methyl-D-aspartate receptor (NMDAR) modulators induce rapid and sustained antidepressant like-activity in rodents through a molecular mechanism of action that involves the activation of Ca^2+^ dependent signaling pathways. Moreover, ketamine, a global NMDAR antagonist is a potent, novel, and atypical drug that has been successfully used to treat major depressive disorder (MDD). However, because ketamine evokes unwanted side effects, alternative strategies have been developed for the treatment of depression. The objective of the present study was to determine the antidepressant effects of either a single dose of hyperforin or lanicemine vs. their combined effects in mice. Hyperforin modulates intracellular Ca^2+^ levels by activating Ca^2+^-conducting non-selective canonical transient receptor potential 6 channel (TRPC6) channels. Lanicemine, on the other hand, blocks NMDARs and regulates Ca^2+^ dependent processes. To evaluate the antidepressant-like activity of hyperforin and lanicemine, a set of *in vivo* (behavioral) and *in vitro* methods (western blotting, Ca^2+^ imaging studies, electrophysiological, and radioligand binding assays) was employed. Combined administration of hyperforin and lanicemine evoked long-lasting antidepressant-like effects in both naïve and chronic corticosterone-treated mice while also enhancing the expression of the synapsin I, GluA1 subunit, and brain derived neurotrophic factor (BDNF) proteins in the frontal cortex. In Ca^2+^ imaging studies, lanicemine enhanced Ca^2+^ influx induced by hyperforin. Moreover, compound such as MK-2206 (Akt kinase inhibitor) inhibited the antidepressant-like activity of hyperforin in the tail suspension test (TST). Hyperforin reversed disturbances induced by MK-801 in the novel object recognition (NOR) test and had no effects on NMDA currents and binding to NMDAR. Our results suggest that co-administration of hyperforin and lanicemine induces long-lasting antidepressant effects in mice and that both substances may have different molecular targets.

## Introduction

Chronic intake, delayed onset of action, drug resistance, and numerous side effects of current antidepressants have forced researchers to look for new and safer drugs with rapid onset and longer acting times (Rosenblat et al., 2015; Sanacora and Schatzberg, 2015). Results obtained in recent years have been encouraging, especially with ketamine, a global NMDAR antagonist. The antidepressant-like activity of ketamine has been shown in many preclinical studies (Maeng et al., 2008; Li et al., 2010, 2011; Autry et al., 2011; Gideons et al., 2014; Miller et al., 2014). A single non-anesthetic dose of ketamine reversed the symptoms of MDD (Berman et al., 2000). The antidepressant effects of ketamine were also observed in patients who suffered from treatment-resistant depression or suicidal ideations (Zarate et al., 2006a; DiazGranados et al., 2010; Murrough et al., 2013; Price et al., 2014). Despite successful clinical trials with ketamine, its use on a large scale is fraught with difficulties and is controversial. Ketamine can induce unwanted side effects such as psychotomimetic symptoms and cognitive disturbances (Rajagopal et al., 2016).

Therefore, other NMDAR antagonists have been tested. Unfortunately, clinical trials with low trapping NMDAR antagonists such as lanicemine (Sanacora et al., 2014, 2017) and other global NMDAR antagonists like memantine (Zarate et al., 2006b) have given inconsistent results and do not show ketamine-like antidepressant potentials. Memantine and lanicemine are also not as effective as ketamine in preclinical studies (Gideons et al., 2014; Qu Y. et al., 2017). However, the data from Preskorn et al. (2008) indicate that NMDA blockade with traxoprodil, a selective antagonist of the GluN2B subunit of the NMDA receptor also produces a robust antidepressant effect. These observations were used to formulate alternative hypotheses that NMDAR blockade could not be the primary molecular mechanism of ketamine action (Zanos et al., 2016, 2017; Collingridge et al., 2017). Moreover, individual enantiomers of ketamine have been shown to activate different intracellular signaling pathways with the induction of different profiles of antidepressant-like activity in mice (Yang et al., 2017, 2018b). Despite all these controversies and doubts surrounding ketamine's primary mode of action, it has been shown that its antidepressant activity in animals is causally dependent on the enhanced processes of neuroplasticity induced in brain regions like the prefrontal cortex (PFC) and hippocampus (Hp) (Li et al., 2010, 2011; Ardalan et al., 2017). At the molecular level, these processes are dependent on the interplay between glutamate receptors, Ca^2+^ channels, intracellular Ca^2+^ levels, Ca^2+^-dependent proteins like Akt, ERK, mTOR, and neurotrophins such as BDNF (Duman and Voleti, 2012; Duman et al., 2016; Workman et al., 2018). The role of Ca^2+^ homeostasis in the antidepressant activity of ketamine was recently reported by Yang et al. (2018a). They showed that the local blockade of NMDAR by ketamine or blockade of the low voltage-sensitive T-type calcium channels (T-VSCC) by mibefradil in the lateral habenula induced antidepressant-like effects in rats (Yang et al., 2018a).

Because Ca^2+^ is the focal point to all the processes mentioned above, we have focused our study on hyperforin, a Ca^2+^-modulator. Hyperforin is the natural and biologically active compound extracted from *Hypericum perforatum* (St John's Wort) (Cervo et al., 2002). Both St John's Wort and hyperforin attenuated symptoms of mild to moderate depression in several clinical trials and displayed antidepressant-like activity in preclinical studies (Zanoli, 2004). Hyperforin also restored cognitive abilities in rats subjected to chronic stress (Liu et al., 2015).

From a molecular perspective, hyperforin has a multi-directional mechanism of action. It blocks conductance of ligand-gated (GABA, NMDA, and AMPA receptors) (Chatterjee et al., 1999; Kumar et al., 2006) and voltage-gated channels (Ca^2+^, K^+^, and Na^+^) (Chatterjee et al., 1999; Fisunov et al., 2000). In contrast to blockade of ion transport through the plasma membrane, hyperforin can generate inward Ca^2+^ currents. *In vitro* studies have shown that hyperforin increases intracellular Ca^2+^ levels by activating TRPC6 or by releasing Ca^2+^ from the mitochondria (Leuner et al., 2007; Tu et al., 2009, 2010). Thus, hyperforin is a potent modulator of intracellular Ca^2+^ levels. Heiser et al. (2013) showed that hyperforin increased the activity of RAS/MEK/ERK and PI3K/Akt or CAMKIV intracellular signaling pathways in PC12 cells and hippocampal CA1 neurons. Gibon et al. (2013) also showed that hyperforin enhanced the expression of TrkB (BDNF receptor), c-AMP response binding-protein (CREB) and the phosphorylated form of CREB (p-CREB) in primary cortical neurons including TrkB in the cortex of adult mice.

It has been shown that *in vitro* (Heiser et al., 2013), hyperforin activates Ca^2+^-dependent signaling pathways involved in neuroplasticity akin to ketamine and NMDAR antagonists, which are known to activate Ca^2+^-dependent signaling pathways *in vivo* (Duman and Voleti, 2012). In addition, Qu et al. recently described a functional relationship between NMDAR and TRPC6. They showed that TRPC6 expression is regulated by NMDAR *in vitro* (Qu Z. et al., 2017). Based on this, we asked the following question: “Can a single dose of hyperforin potentiate antidepressant-like activity a single dose of NMDAR antagonists in mice and what is the mechanism of action?”

To answer this question we selected two NMDAR antagonists: lanicemine and MK-801. In the first phase of the study, we determined whether there was an interaction between NMDAR and hyperforin using TST in naïve mice. Secondly, we determined the effects of the combined administration of a single dose of hyperforin and lanicemine in mice exposed to chronic corticosterone treatment.

Because hyperforin's potential to improve cognitive activity has been described elsewhere (Klusa et al., 2001; Liu et al., 2015), in the next part of our study we evaluated hyperforin's potential to attenuate cognitive deficits induced by MK-801 in the NOR test. This is a very important area of research, because ketamine and other NMDAR antagonists can induce cognitive deficits (Rajagopal et al., 2016) which are also present in MDD (Lam et al., 2014), Next, we determined whether hyperforin's antidepressant-like activity was dependent on select Ca^2+^signaling pathways and the glutamate system. The last phase of the study was devoted to determining the potential biochemical and electrophysiological mechanisms induced by hyperforin and lanicemine.

## Materials and Methods

### Animals

Male and female adult (9–10 weeks, 23–25 g) C57BL/6J mice (Charles River) were used in the experiments. Mice were housed under a natural 12 h light/dark cycle in a room with controlled temperature with *ad libitum* access to water and food. All behavioral experiments were performed between 9 a.m. and 2 p.m. All studies were performed according to the guidelines of the European Community Council (Directive 86/609/EEC) and were approved by the Ethical Committee of the Institute of Pharmacology.

### Compounds and Treatment

All the compounds/drugs used in the study were obtained commercially except hyperforin (sodium salt), which was a gift from Dr. Wilmar Schwabe GmbH & Co (Karlshrue Germany). Doses of compounds were chosen based on results from our preliminary studies (lanicemine and hyperforin) or from literature (MK-801, fluoxetine, NMDA). Pertinent information on the various compounds is presented in Table 1.

**Table 1:** Pertinent information on the various compounds/drugs.

**Compound**	**Hyperforin sodium salt**	**Lanicemine**	**MK-2206**	**MK-801**	**Larixyl acetate**	**NMDA**	**Fluoxetine**
Doses mg/kg	1, 2.5; 5; 10 *i.p*.	2, 10 *i.p*.	20 *i.p*.	0.1; 0.3 *i.p*.	10 nM/2 μL *i.c.v*	75 *i.p*.	10 *i.p*.
Solvent	Aqua pro injection	Aqua pro injection	10% DMSO	Aqua pro injection	Phosphate buffer + 1% DMSO	Aqua pro-injection	Aqua pro injection
Source	Wilmar Schwabe	Sigma Aldrich	Selleckchem	Sigma Aldrich	Sigma Aldrich	Sigma Aldrich	Selleckchem

*i.p., intraperitoneal; i.c.v, intracerebroventricular*.

### Cannulae Implantation

For intracerebroventricular (*i.c.v*.) injection of compounds, mice were anesthetized with ketamine (100 mg/kg i.p.) and xylazine (10 mg/kg i.p.; Biowet, Poland) and stereotaxically, bilaterally implanted with the guide cannulae (8 mm); (coordinates relative to bregma: 1 mm lateral, 0.2 mm posterior and 3,7 mm ventral). After 14 days of recovery, mice were subjected to injection and behavioral studies. Compounds were applied to each ventricle for 1 min, followed by a 1 min diffusion time. Mice received one injection each through both ventricles (1 μL per ventricle) using an infusion cannula (9 mm) 15 min before *i.p*. hyperforin (2.5 mg/kg) administration. 1 h after hyperforin treatment TST was conducted.

### Chronic Exposure to Corticosterone

Male C57BL/6J mice (10 weeks old) which included controls and Cort-treated (Cort) groups were housed 6 per cage. The Cort group received corticosterone (25 μg/ml) in drinking water for the first 4 weeks. Corticosterone (Sigma Aldrich) was dissolved in concentrated ethanol and added to the drinking water to give a final concentration of 0.5%. Controls, only received 0.5% ethanol in drinking water. All bottles were wrapped in foil and replaced every 3 days. In the 5th week, corticosterone concentration was gradually reduced as follows: Cort group received 12.5 μg/ml of corticosterone in the first 3 days followed by 6.25 μg/ml in the next 3 days.

Animals were Cort free during the next 7 days, after which behavioral tests were carried out. All behavioral tests were performed on the same mice. Administration of Cort in drinking water has been described in several papers (Gourley et al., 2008; Miller et al., 2014; Zanos et al., 2016).

### Forced Swim Test

The Forced swim test (FST) was conducted as previously described (Szewczyk et al., 2010). Mice were put individually in a glass cylinder (10 cm diameter, 25 cm high) filled with water (23–25°C) to the height of 10 cm for 6 min. Following that, immobility time was measured in the last 4 min of the experiment. Mice that remained floating passively were judged to be immobile.

### Tail Suspension Test

TST was conducted as previously described (Steru et al., 1985). Briefly, each mouse was individually suspended by the tail, using adhesive tape (2 cm from the tail tip) glued to a solid flat surface. Immobility time was measured for 6 min. A mouse was judged to be immobile when it was hanging passively and completely motionless.

### Locomotor Activity

Evaluation of the specificity of the effects observed in the FST and TST was assessed by locomotor activity (Table 2). Plexiglas locomotor activity chambers (40 × 20 × 15 cm) in a 20-station photo-beam activity system (Columbus Instruments, Opto-M3-activity meter) were used to measure locomotor activity. Each mouse was placed individually in the chamber for a 6 min session during which the total number of ambulations was measured.

**Table 2:** The effect of used compounds and procedures on the locomotor activity of mice.

	**Treatment mg/kg**	**Locomotor activity (% of control)**
A.	Control Lanicemine 2 Lanicemine 2 + Hyperforin 1 Lanicemine 10 Lanicemine 10 + Hyperforin 5	100.0 ± 5.46 105.1 ± 9.16 129.2 ± 13.88 87.77 ± 4.70 82.8 ± 12.96
B.	Control MK-801 MK-801 + Hyperforin	100.0 ± 4.89 123.8 ± 15.24 76.44 ± 12.29^**#**^
C.	Control Hyperforin 2.5 Lanicemine Fluoxetine Lanicemine + Hyperforin CORT CORT + Hyperforin CORT + Lanicemine CORT + Fluoxetine CORT + Lanicemine+Hyperforin	100.0 ± 7.21 91.74 ± 8.12 131.1 ± 13.97 104.2 ± 7.87 90.74 ± 12.2 122.9 ±6.45 90.88 ±9.60 130.2 ± 15.71 97.94 ± 2.35 101.6 ± 11.69
D.	Control Hyperforin 5 NMDA 75 NMDA 75 + Hyperforin 5 MK 2206 MK 2206 20 + Hyperforin 5	100.0 ± 3.16 55.83 ± 5.72^**^ 69.25 ± 8.00^*^ 57.01 ± 11.45^**^ 82.99 ± 6.98 73.4 ± 6.67^*^
E.	Control Larixyl acetate 10nmol/2ul Hyperforin 2.5 Larixyl acetate 10nmol/2ul + Hyperforin 2.5	100.0 ± 9.60 84.5 ± 9.51 87.97 ± 21.6 40.31 ± 10.81^*^

*Locomotor activity was measured immediately after TST*.

* *p < 0.05*;

** *p < 0.01 vs. Control*;

# *p < 0.05 vs. MK-801. Data was analyzed by one way ANOVA **(A–D)** or two way ANOVA and Newman-Keuls multiple comparisons test. All values are expressed as mean ± S.E.M. **(A)** F_(4, 20)_ = 3.719; p = 0.02; **(B)** F_(2, 12)_ = 4.191; p = 0.04; **(C)** [CORT: F_(1, 47)_ = 0.659; p = 0.421; Treatment F_(4, 47)_ = 4.476; p = 0.004; Interaction F_(4, 47)_ = 0.716; p = 0.585]. **(D)** F_(5, 28)_ = 5.668; p = 0.001; **(E)** F_(3, 13)_ = 3.766; p = 0.038. n = 4–6*.

### Splash Test

A Splash test was conducted as described by Yalcin et al. (2008). Briefly, 10% sucrose solution was sprayed on the back of each mouse. The viscous nature of the solution induces enhanced grooming behavior. Each session lasted 5 min with grooming behavior measured accordingly.

### Novel Object Recognition Test

This test was conducted as previously described (Wozniak et al., 2016). In brief, training, habituation and test sessions took place in a black plastic rectangular open field (50 × 30 × 35 cm) illuminated by a 25 W bulb. Habituation was carried out for 10 min for 2 days during which each mouse was placed individually in the open field in the absence of objects and allowed to explore the environment. Training and test sessions were performed 24 h after habituation. In the first session (5 min) mice explored two identical objects (red glass cylinders 6.6 cm in diameter and 4.5 cm high). During the second session (1 h later), the familiar object was replaced by a novel object (a transparent elongated sphere-like object with an orange cap, 5.5 cm in diameter, 8.5 cm high). Mice were allowed to explore this environment for 5 min. They were administered hyperforin (30 min interval) followed by MK-801 which was administered 30 min before the first training session. Time spent exploring (i.e., sniffing or touching) the familial (T familial) or novel object (T novel) was measured by a trained observer followed by calculation of the recognition index [(T familial–T novel)/(T familial + T novel)].

### Western Blotting

Western blotting was conducted as previously described (Szewczyk et al., 2014; Rafalo et al., 2017). Briefly, after decapitation, the frontal cortex was rapidly dissected from each mouse, frozen on dry ice and stored at −80°C. Next, tissue was homogenized in a 2% solution of sodium dodecyl sulfate (SDS), denatured at 95°C for 10 min and centrifuged for 5 min at 10,000 rpm. The protein in the supernatant was assayed by the bicinchoninic acid method (Pierce). Proteins were fractionated on a 10 or 12% SDS polyacrylamide gel and transferred to nitrocellulose membrane (Bio-Rad). Non-specific binding was blocked by 1% blocking solution (BM Chemiluminescence Western Blotting Kit Mouse/Rabit, Roche). Following blocking, membranes were incubated overnight with the following antibodies: BDNF (~15 kD, 1:500, Monoclonal, Santa Cruz), phosphorylated CREB (p-CREB); (~43 kD, 1:1,000, monoclonal, Millipore), GluA1-AMPA (~100 kD, 1:1,000, polyclonal, Abcam), Synapsin I (~74 kD, 1:1,000, monoclonal, Abcam,), total CREB (~43 kD, 1:1,000. monoclonal, Santa Cruz), β-actin (~42 kD, 1:10,000, monoclonal, Sigma). The next day, membranes were washed (3 times) with Tris-buffered saline containing Tween (TBST) and incubated with secondary mouse/rabbit antibodies (1:7,000, Roche). Finally, blots were washed 3 times in TBST and incubated in the detection reagent (Roche). The signal from each protein was measured and visualized using the Fuji-Las 1000 system and Image Gauge v 4.0. β-actin was used as a loading control and for normalization (Figure S4). The densities of the bands obtained for phosphorylated and total CREB were first normalized to appropriate actin bands and then the ratio of normalized p-CREB/CREB was calculated. Data on the graph are expressed as % of change vs. control.

### Whole Cell Patch Clamp Studies

#### Brain Slice Preparation for Patch Clamp Experiments

Mice were decapitated under isoflurane anesthesia (Aerrane, Baxter) between 9 and 10 a.m. Brains were quickly removed and placed in ice-cold artificial cerebrospinal fluid (aCSF) containing (in mM): 130 NaCl, 5 KCl, 2.5 CaCl_2_, 1.3 MgSO_4_, 1.25 KH_2_PO_4_, 26 NaHCO_3_, and 10 D-glucose. Coronal slices (300 μm) containing the mPFC were cut using a vibrating microtome (Leica VT1000) and subsequently incubated in carbonated aCSF at 30 ± 0.5°C for at least 1 h before recording.

#### Whole-Cell Patch Clamp Recording

To record NMDA currents, slices were placed in the recording chamber superfused at 3 ml/min with warm (32 ± 0.5°C), modified Mg^2+^-free aCSF with the following composition (in mM) 132 NaCl, 2 KCl, 2.5 CaCl_2_, 1.25 KH_2_PO_4_, 26 NaHCO_3_, 10 D-glucose, continuously bubbled with a mixture of 95% O_2_ and 5% CO_2_. NBQX (5 μM) was added to block AMPA/kainate receptors. Neurons were visualized using a Zeiss AxoExaminer.A1 upright microscope equipped with IR DIC optics, a 40 × water immersion lens and an infrared camera (Sony). Patch pipettes pulled from borosilicate glass capillaries (Harvard Instruments) using a Sutter Instrument P97 puller had open tip resistances of approximately 3–5 MΩ when filled with a solution containing (in mM): 130 K-gluconate, 5 NaCl, 0.3 CaCl_2_, 2 MgCl_2_, 10 HEPES, 5 Na_2_-ATP, 0.4 Na-GTP, and 1 EGTA. Osmolarity and pH were adjusted to 290 mOsm and 7.2, respectively. Signals were recorded with the Multiclamp 700B amplifier (Molecular Devices), filtered at 2 kHz and digitized at 20 kHz using the Digidata 1,550 interface and pCLAMP 10 software (Molecular Devices).

#### Stimulation and Recording of NMDA Postsynaptic Currents

Synaptic NMDA currents were evoked using a concentric platinum/stainless steel electrode (FHC, USA) by stimulating (every 30 s, duration 0.2 ms) layer V of the mPFC while recording from mPFC layer II/III pyramidal neurons in a voltage clamp mode with a holding potential of −70 mV. Stimulation intensity was adjusted so that a stable NMDA current of approximately 100 pA could be recorded for at least 10 min before drug application.

Currents were percentage-normalized with reference to 5 min of the recording right before drug application. Percentage of the response was measured by averaging 5 consecutive currents after 10 min of drug application. At this time the response to lanicemine (2 μM) treatment plateaued while the response to hyperforin (0.5 μM) remained stable.

### Extracellular Field Potential Recordings

Mice were decapitated, their frontal cortices were dissected and cut into 400 μm-thick slices which were stored in a gassed (95% O_2_ and5% CO_2_) ACSF, consisting of (in mM): 127 NaCl, 5 KCl, 2.5 CaCl_2_, 1.3 MgSO_4_, 1.25 KH_2_PO_4_, 24NaHCO_3_, and 10 glucose. Individual slices were placed in the recording chamber of an interface type which was superfused (2.5 ml/min) with a modified ACSF, (temperature 32.0 ± 0.5°C) containing (in mM) NaCl (132), KCl (2), CaCl_2_ (2.5), KH_2_PO_4_ (1.25), NaHCO_3_ (26), and D-glucose (10), bubbled with 95% O_2_ and 5% CO_2_ (temperature 32.0 ± 0.5°C). To study the NMDA receptor-mediated component of field potentials (FP), the slices were perfused with ACSF devoid of Mg^2+^ ions and supplemented with 5 μm of 2,3-dioxo-6-nitro-1,2,3,4-tetrahydrobenzo[f]quinoxaline-7-sulfonamide (NBQX, Tocris Bioscience), an AMPA receptor antagonist.

A concentric bipolar stimulating electrode (FHC, USA) was placed in cortical layer V. The recording electrode was placed in layers II/III of the mPFC (prelimbic cortex region). Stimuli of 0.033 Hz frequency and duration of 0.2 ms were applied using a constant-current stimulus isolation unit (WPI). Glass micropipettes filled with ACSF (2–5 MΩ) were used to record field potentials. The responses were amplified (EXT 10–2 F amplifier, NPI), filtered (1 Hz−1 kHz), A/D converted (10 kHz sampling rate), and stored on PC using the Micro1401 interface and Signal 4 software (CED).

After the 30 min, of incubation and after stabilizing the responses, a stimulus was adjusted to evoke a response of 40% of the maximum amplitude. After 15 min of baseline recording a hyperforin 1 uM was added (20 min) and recording was continued for 70 min.

### Calcium Imaging

Primary cultures of cortical neurons were prepared from embryonic (E13) C57BL6/J mice as previously described (Bouron et al., 2005) and approved by the animal care committee of the CEA's Life Sciences Division (CEtEA). Experiments were conducted in accordance with French legislation and the European Community Council Directive of 24 November 1986 (86/609/EEC). Calcium imaging experiments were performed at room temperature as previously described (Gibon et al., 2013; Chauvet et al., 2015, 2016). Briefly, cortical neurons were incubated in a saline solution containing (mM) 150 NaCl, 5 KCl, 1 MgCl_2_, 2 CaCl_2_, 5.5 glucose, 10 HEPES (pH 7.4) supplemented with 5 μM Fluo4/AM. After 20 min of incubation, neurons were rinsed twice and incubated for 10 min in a Fluo-4/AM-free saline. The imaging system consisted of an inverted Axio Observer A1 microscope equipped with a Fluar 40 × oil immersion objective lens (1.3 NA) (Carl Zeiss, France) and a CCD CoolSnap HQ2 camera (Princeton Instruments, Roper Scientific, France). A DG-4 wavelength switcher (Princeton Instruments, Roper Scientific, France) was used with λ_EX_ = 470 nm and λ_EM_ = 525 nm. The setup was driven by MetaFluor (Universal Imaging, Roper Scientific, France).

### Radioligand Binding Studies

Radioligand binding assay was performed according to the method of (Fischer et al., 1997) with slight modifications. Binding experiments were conducted in 96-well microplates in a total volume of 300 μL. The reaction mix included 30 μL amounts of test compounds, 30 μL of radioligand and 240 μL of tissue suspension. Tissue (rat cortex) was homogenized in 50 volumes of ice-cold 50 mM Tris-HCl buffer with 10 mM ethylenediaminetetraacetic acid, pH 7.4 using an Ultra Turrax T25B (IKA) homogenizer. The homogenate was centrifuged at 35,000 × g for 15 min. The resulting pellet was resuspended in the same quantity of buffer and centrifuged two more at the same speed and frozen at −80°C for at least 16 h and not more than 2 weeks. For binding experiments, the membranes were washed three times (homogenization in 25 volumes of cold 5 mM Tris-HCl (pH 7.4) with an Ultra-Turrax at maximum speed for 30 s). The final pellet was resuspended in an appropriate volume of buffer (10 mg/1 ml) for use in the assay. Incubation was performed in the presence of 10 μM added glutamate and glycine. The ligand, [^3^H]-MK-801 (spec. act. 40 Ci/mmol, Perkin Elmer) was used at a final concentration of 5 nM. Non-specific binding was determined in the presence of 10 μM MK-801. After a 2 h-incubation at room temperature, the reaction mix was filtered immediately onto GF/B filter mate. Ten rapid washes were performed with chilled 5 mM Tris pH 7.4 buffer, using an automated harvesting system Harvester-96 MACH III FM (Tomtec, USA). Filter mates were dried at 37°C in a forced air fan incubator CLW 32 STD (Pol-Eko Aparatura, Poland) and then solid scintillator was melted onto the filter mates at 100°C for 5 min. The radioactivity retained on the filter was counted in a MicroBeta2 LumiJET scintillation counter (PerkinElmer, USA). The compounds analyzed ranged in concentration from 10^−10^ to 10^−3^M. All assays were done in duplicates.

### Statistical Analysis

Statistical analysis of behavioral and Western blot studies was performed using GraphPad Prism. We used one or two way ANOVA when appropriate, followed by the Newman-Keuls *post-hoc* test. Statistical significance was set at *p* < 0.05.

Analysis of recorded NMDA currents was carried out using the pCLAMP 10 and Graphpad Prism software programs. The obtained NMDA current values were analyzed using a one-sample Wilcoxon Signed Rank Test against 100% separately for hyperforin- and lanicemine.

Radioligand binding data were analyzed using iterative curve fitting routines GraphPAD/Prism – San Diego, CA, USA). Ki values were calculated from the Cheng and Prusoff (1973) equation.

Calcium imaging data were analyzed by Student's *t*-test.

Extracellular field potential recordings were analyzed by Wilcoxon matched-pairs signed rank test.

## Results

### Hyperforin Potentiates the Effects of Lanicemine and MK-801 in the TST

**Figure 1:**
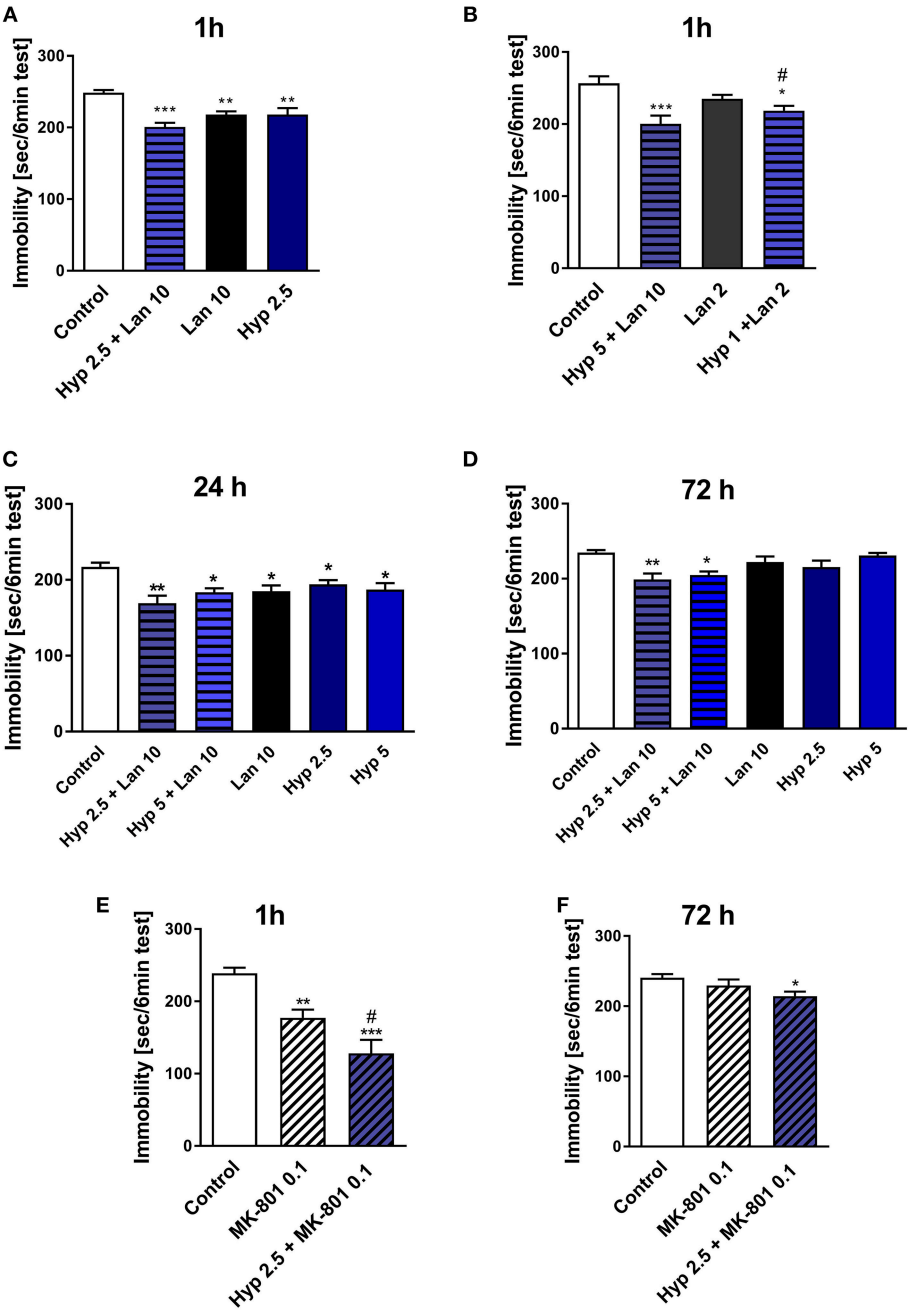
The effect of combined administration of a single dose of hyperforin (Hyp) and NMDAR antagonists: lanicemine (Lan) and MK-801 in TST in mice. **(A,B)** Lanicemine (2 or 10 mg/kg; *i.p*.) was administered 90 min before the TST and 30 min before hyperforin (1, 2.5, or 5 mg/kg; *i.p*.) treatment. ^***^p < 0.001 vs. control, ^**^p < 0.01 vs. control, ^*^p < 0.05 vs. control, ^#^*p* < 0.05 vs. lanicemine 10 + Hyp 5, *n* = 7–8; [A: *F*_(3, 22)_ = 10.65; *p* = 0.0002], [B: *F*_(3, 22)_ = 7.405; *p* = 0.0013]. **(C,D)** Hyperforin and lanicemine were administered in the same way as in **(A,B)** but the TST was carried out 24 h **(C)** or 72 h **(D)** after hyperforin treatment. ^**^p < 0.01 vs. control, ^*^p < 0.05 vs. control, *n* = 7–8; [C: *F*_(5, 38)_ = 4.376; *p* = 0.003], [D: *F*_(5, 39)_ = 5.063; *p* = 0.0011]. **(E,F)** MK-801 (0.1 mg/kg; *i.p*.) was administered 30 min before hyperforin (2.5 mg/kg; *i.p*.), 1 h **(E)** or 72 h **(F)** after hyperforin treatment TST was carried out. ^***^p < 0.001 vs. control, ^**^p < 0.01 vs. control, ^#^*p* < 0.05 vs. MK-801, *n* = 7–8; [E: *F*_(2, 21)_ = 17.20; *p* = 0.0001], [F: *F*_(2, 19)_ = 3.696; *p* = 0.0441]. All data was analyzed by one-way ANOVA and Newman-Keuls multiple comparisons test. Values are expressed as mean ± S.E.M.

As mentioned earlier, the functional relationship between NMDAR and TRPC6 receptor has been described previously (Qu Z. et al., 2017). Hyperforin and NMDAR antagonists' antidepressant activity have also been previously reported (Zanoli, 2004; Sanacora and Schatzberg, 2015). Both hyperforin and NMDAR antagonists activate similar Ca^2+^-dependent intracellular processes (Duman and Voleti, 2012; Heiser et al., 2013). Therefore, we decided to investigate whether the combined administration of hyperforin and lanicemine or MK-801 can provide some beneficial antidepressant effects (stronger effects or long-lasting activity). We choose lanicemine and MK-801 two structurally different NMDAR antagonists. In this part of our studies, we examined the antidepressant-like activity of single active doses of hyperforin (2.5 and 5 mg/kg), lanicemine (10 mg/kg) and a combined administration of hyperforin (2.5 or 5 mg/kg) and lanicemine (10 mg/kg) at three different time points; 1, 24, and 72 h after treatment. Additionally, we evaluated the effects of the combined administration of non-active-doses of hyperforin (1 mg/kg) and lanicemine (2 mg/kg) 1 h after treatment. Both at the 1 and 24 h time points all drugs evoked antidepressant activity (Figures 1A–C). Also, the administration of a combined non-active dose of lanicemine (2 mg/kg) and non-active dose of hyperforin (1 mg/kg) induced antidepressant-like activity after 1 h (Figure 1B). At the 72 h time point, hyperforin and lanicemine administered alone did not evoke any antidepressant effects, but a combined treatment with hyperforin and lanicemine significantly reduced the immobility time in the TST (Figure 1D).

In our preliminary studies combined treatment with hyperforin (2.5 mg/kg) + lanicemine (10 mg/kg) significantly reduced the immobility time in female mice both at the 1 h (TST) and 72 h (FST) time points (Figure S1).

The effects observed in the TST were not associated with increased locomotor activity (Table 2A). Moreover, hyperforin (2.5 mg/kg) potentiated short-lasting antidepressant effects induced by active dose of MK-801 (0.1 mg/kg) (Figure 1E). A single dose of MK-801 did not evoke any effects in the TST 72 h after administration. In contrast, a combined administration of hyperforin and MK-801 evoked an antidepressant response (Figure 1F). MK-801 and hyperforin alone had no effect on locomotor activity. However, a combined administration of MK-801 and hyperforin decreased locomotor activity compared to the MK-801 group (Table 2B).

### Combined Treatment of a Single Dose of Hyperforin and Lanicemine Reversed Behavioral Disturbances in Mice Induced by Chronic Corticosterone Administration

**Figure 2:**
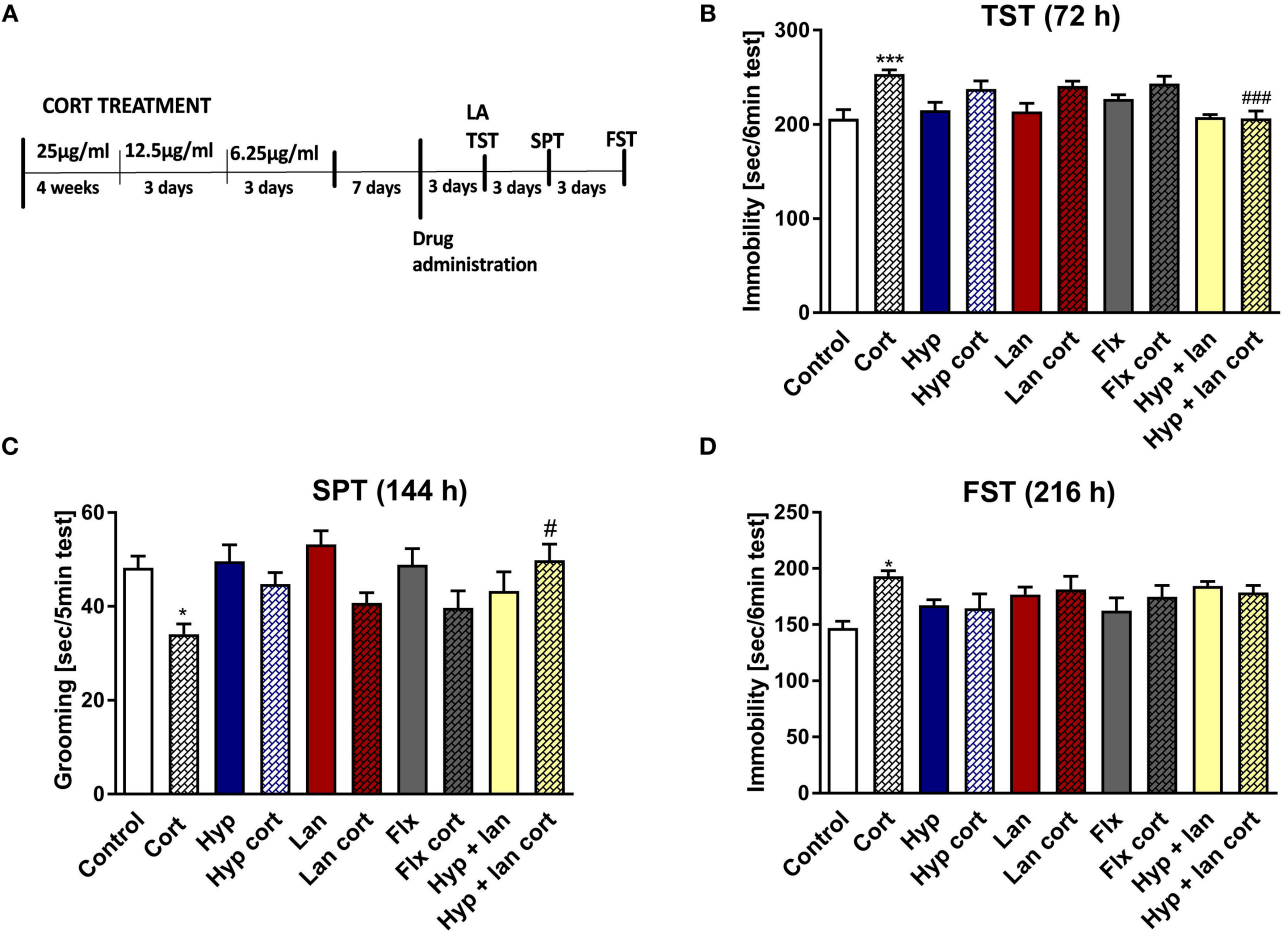
The effect of the administration of single doses of hyperforin (Hyp), lanicemine (Lan), fluoxetine (Flx), and hyperforin + lanicemine (Hyp + Lan) on behavioral disturbances induced by chronic corticosterone (Cort) treatment *n* = 9–11. Single doses of the following drugs: hyperforin (2.5 mg/kg; *i.p*.), lanicemine (10 mg/kg; *i.p*.), fluoxetine (10 mg/kg; *i.p*.) were used. **(A)** Experimental schedule of drug treatments and behavioral tests; **(B)** TST was carried out 72 h after treatment. ***p < 0.001 vs. control, ^###^*p* < 0.001 vs. cort. Two-way ANOVA showed cort effect [*F*_(1, 91)_ = 24.42; *p* = 0.0001], treatment effect [*F*_(4, 91)_ = 4.55; *p* = 0.002], and interaction effect [*F*_(4, 91)_ = 3.248; *p* = 0.015]. **(C)** Splash test (SPT) was done 144 h after treatment. *p < 0.05 vs. control, ^#^*p* < 0.05 vs. cort. Two-way ANOVA showed Cort effect [*F*_(1, 91)_ = 12.725; *p* = 0.00057], no treatment effect [*F*_(4, 91)_ = 1.252; *p* = 0.294] and interaction effect [*F*_(4, 91)_ = 3.058; *p* = 0.0205]. **(D)** FST was carried out 216 h after treatment. *p < 0.05 vs. control, two-way ANOVA showed cort effect [*F*_(1, 91)_ = 4.398; *p* = 0.03875], no treatment effect [*F*_(4, 91)_ = 1.84; *p* = 0.24578] and interaction effect [*F*_(4, 91)_ = 3.412; *p* = 0.012]. All data was analyzed by two-way ANOVA and Newman-Keuls multiple comparisons test. All values are expressed as mean ± S.E.M.

Because the TST is a simple behavioral screening test, in next phase of the studies we wanted to find out if the beneficial antidepressant-like activity of hyperforin and lanicemine seen in the TST also occurs under complex conditions such as those evoked by chronic corticosterone administration which mimics stressful conditions where corticosterone is released in response to stressful stimuli (Wilner, 2017). The hypothalamic pituitary adrenal (HPA) axis is the central stress response system. Disturbances in the functioning of the HPA axis have been described in MDD patients and in mice subjected to chronic stress (Keller et al., 2017; Wilner, 2017). We examined the effects of combined administration of active doses of hyperforin and lanicemine, lanicemine, hyperforin, or fluoxetine given to control and Cort-treated mice. Doses of hyperforin and lanicemine were selected on the basis of the respective activity of each drug in the TST in naïve mice. Single doses of hyperforin (2.5 mg/kg), lanicemine (10 mg/kg), fluoxetine (10 mg/kg) and lanicemine (10 mg/kg) + hyperforin (2.5 mg/kg) were administered 7 days after Cort withdrawal. The TST and splash test were conducted 72 and 144 h, respectively, after drug administration (Figure 2A). Cort-treated mice displayed increased immobility times compared to controls in the TST (Figure 2B). Administration of hyperforin, lanicemine, and fluoxetine did not reverse these effects. However, a combined treatment with hyperforin and lanicemine abolished these effects (Figure 2B). In the splash test, Cort-treated mice showed decreased grooming time compared to controls (Figure 2C). This depressive-like behavior was reversed only by a combined treatment of hyperforin and lanicemine (Figure 2C). Next, 9 days (216 h) after drug administration, FST was performed (Figure 2D). We observed increased immobility times in Cort-treated mice and no effects of the antidepressant treatment strategies (Figure 2D). Effects observed in the TST and FST were specific because changes in locomotor activity were not noticed (Table 2C).

### Hyperforin Reversed MK-801 Induced Effects in the Novel Object Recognition Test

**Figure 3:**
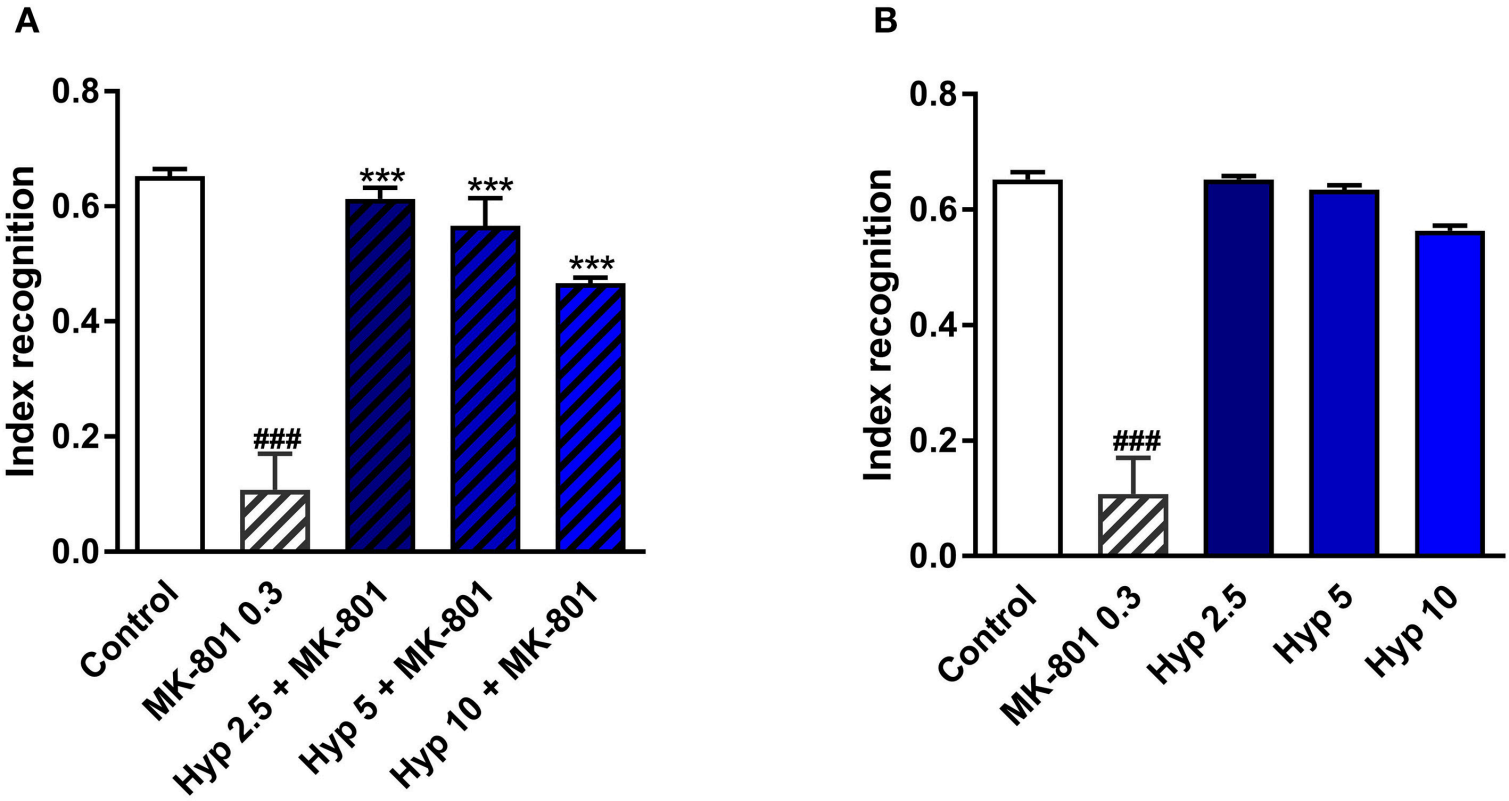
The effect of pretreatment with glutamate system modulator on hyperforin activity in the NOR in mice. **(A,B)** Hyperforin (2.5, 5, 10 mg/kg; *i.p*.) was administered 60 min before the first session. MK-801 (0.3 mg/kg; *i.p*.) was administered 30 min before the first training session of the NOR test. ^###^*p* < 0.001 vs. control, ***p < 0.001 vs. MK-801, *n* = 6–8; [**A**: *F*_(4, 32)_ = 70.04; *p* = 0.0001], [**B**: *F*_(4, 30)_ = 36.08; *p* = 0.0001]. All data was analyzed by one-way ANOVA and Newman-Keuls multiple comparisons test. All values are expressed as mean ± S.E.M.

NMDAR antagonists, including ketamine, have been shown to induce some cognitive impairment (Rajagopal et al., 2016). Cognitive deficits have also been identified in patients diagnosed with MDD (Lam et al., 2014). Therefore, the improvement of cognitive function is a very desirable feature for any novel or potential antidepressant. The procognitive effects of hyperforin have been previously reported (Klusa et al., 2001; Liu et al., 2015). In the present studies, a single dose of hyperforin (2.5, 5, 10 mg/kg) reversed cognitive disturbances in mice as measured by the cognitive index evoked by a single dose of MK-801 (0.3 mg/kg) in the NOR test (Figure 3A). Hyperforin, administered alone did not have any effects on the recognition index (Figure 3B).

### Pretreatment With N-Methyl-D-Aspartic Acid Abolished Antidepressant-Like Activity of Hyperforin

**Figure 4:**
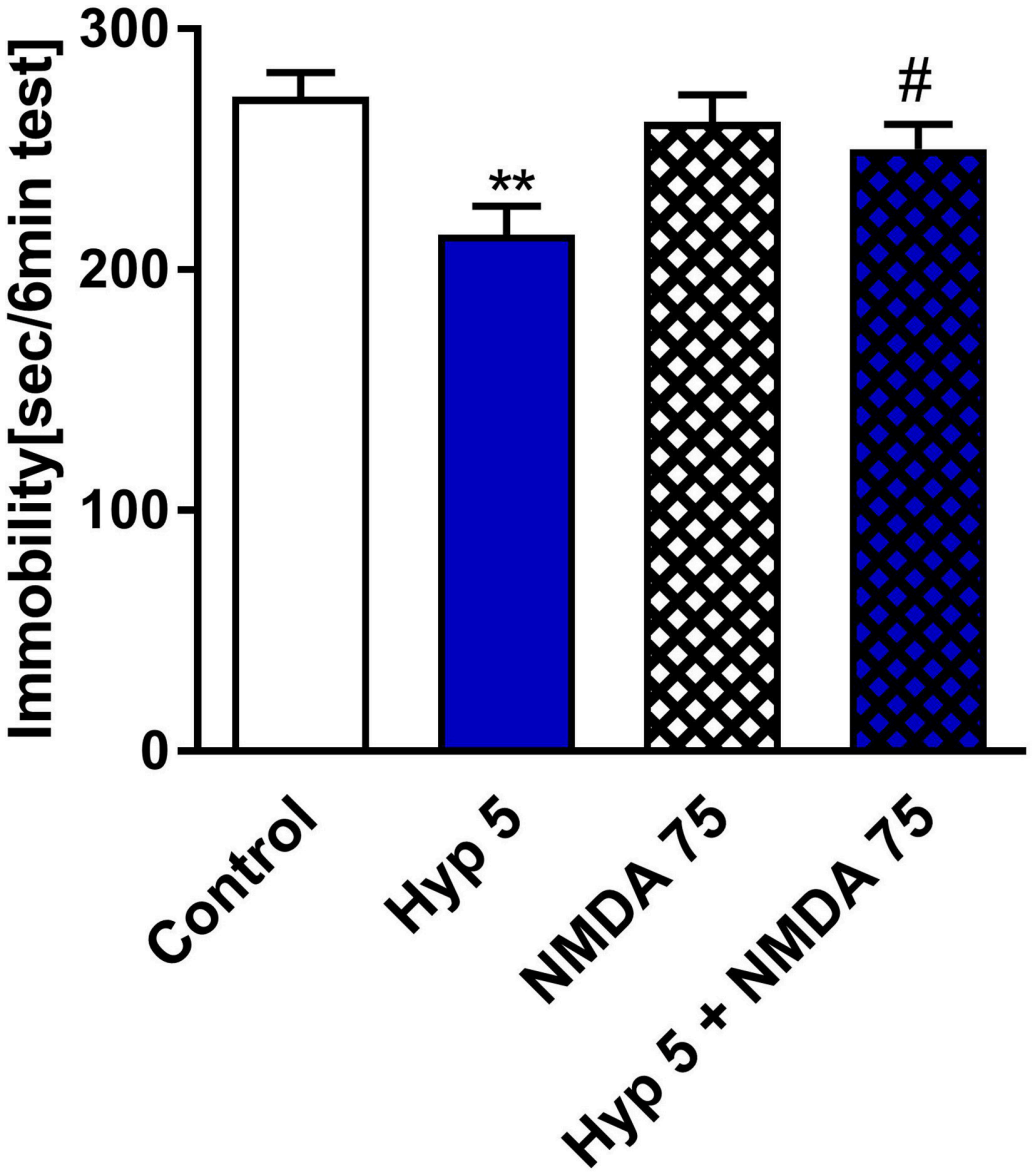
The effect of pretreatment with glutamate system modulator on hyperforin activity in the TST in mice. NMDA (75 mg/kg; *i.p*.) was administered 15 min before hyperforin (2.5 mg/kg; *i.p*.). TST was carried out 60 min after hyperforin administration. **p < 0.01 vs. control, ^#^*p* < 0.05 vs. hyperforin, *n* = 5–7; [*F*_(3, 22)_ = 4.957; *p* = 0.0089]. All data was analyzed by one-way ANOVA and Newman-Keuls multiple comparisons test. All values are expressed as mean ± S.E.M.

A few *in vitro* studies have shown that hyperforin can block the NMDAR (Chatterjee et al., 1999; Kumar et al., 2006). Thus, we pretreated mice with NMDA and monitored them for changes in the behavioral response to hyperforin in the TST. Figure 4 shows that hyperforin (5 mg/kg) induced antidepressant-like activity in the TST which was abolished by the pretreatment with NMDA (75 mg/kg/body weight). The NMDA dose used was selected based on previous studies (Poleszak et al., 2007; Wolak et al., 2013). Also, NMDA/CNS related responses after *i.p*. administration have been described by Budziszewska et al. (1998). Hyperforin given alone and in combination with NMDA decreased locomotor activity in comparison to control group. Therefore, decreased locomotor activity is not related to NMDA co-administration (Table 2D).

### Administration of Larixyl Acetate and MK-2206 Abolished Effects of Hyperforin Observed in the TST

**Figure 5:**
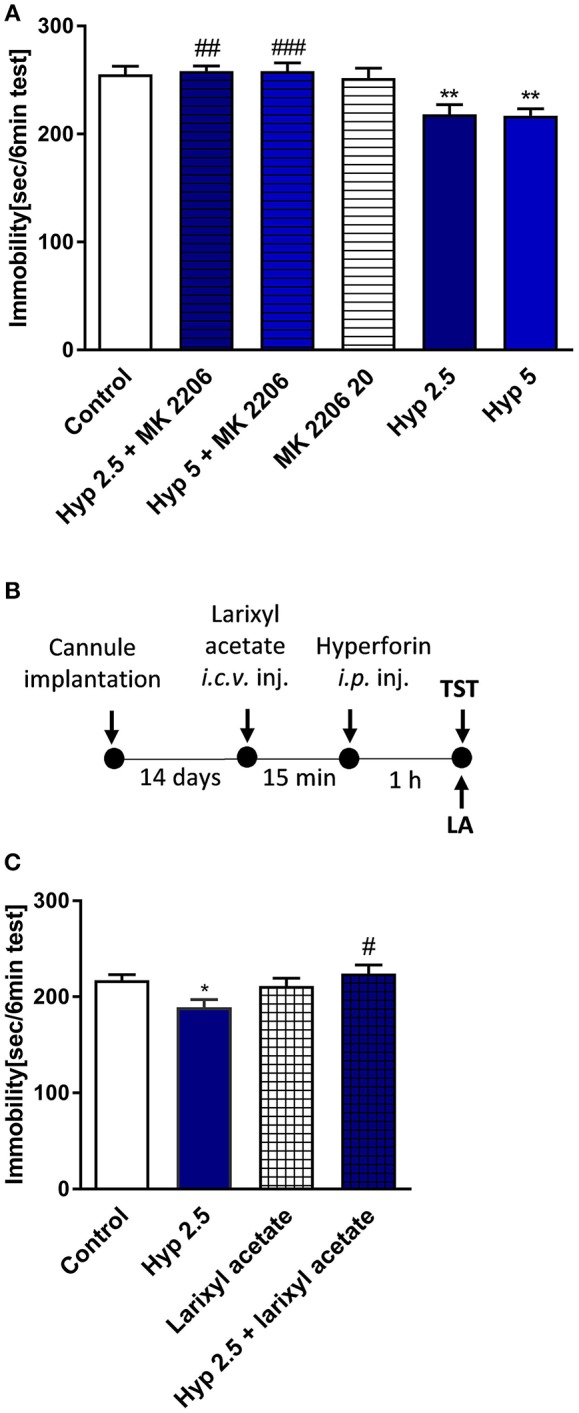
The effect of pretreatment with Ca^2+^-dependent process modulators on hyperforin activity in the TST in mice **(A)** MK-2206 (Akt1/2/3 kinase inhibitor: 20 mg/kg; *i.p*.) was administered 15 min before hyperforin (2.5 and 5 mg/kg; *i.p*.). TST was carried out 60 min after hyperforin treatment. **p < 0.01 vs. control, ^##^*p* < 0.01 vs. hyperforin 2.5, ^###^*p* < 0.001 vs. hyperforin 5, *n* = 6–8; [*F*_(5, 34)_ = 7.144; *p* = 0.001]. **(B)** Experimental schedule of drug treatments and behavioral tests. **(C)** Larixyl acetate (TRPC6 antagonist: 10 nM/2 μL; *i.c.v*.) was injected 15 min before hyperforin (2.5 mg/kg; *i.p*.) and 60 min after hyperforin treatment the TST was carried out *p < 0.05 vs. control, ^#^*p* < 0.05 vs. hyperforin, *n* = 9–10; [*F*_(3, 35)_ = 3.892; *p* = 0.0168]. All data was analyzed by one-way ANOVA and Newman-Keuls multiple comparisons test. All values are expressed as mean ± S.E.M.

Results obtained from previous *in vitro* studies indicated that hyperforin-induced cellular effects are Ca^2+^-dependent (Leuner et al., 2007; Tu et al., 2009, 2010), thus we wanted to find out whether Ca^2+^-dependent processes are involved in the behavioral response of hyperforin. In particular, TRPC6 channels and AKT kinase are thought to be involved in these effects. Preinjection with larixyl acetate a potent TRPC6 antagonist (Urban et al., 2016) (10 nM/2 μL) and pretreatment with Akt 1/2/3 kinase inhibitor - MK-2206 (Cheng et al., 2012) (20 mg/kg) abolished the hyperforin induced effects in the TST (Figures 5A–C). However, these results should be interpreted carefully because hyperforin + larixyl acetate induced decreased locomotor activity when compared to hyperforin group (Table 2E).

### Hyperforin Does Not Affect NMDAR Synaptic Currents and NMDA Component of the Field Potential

**Figure 6:**
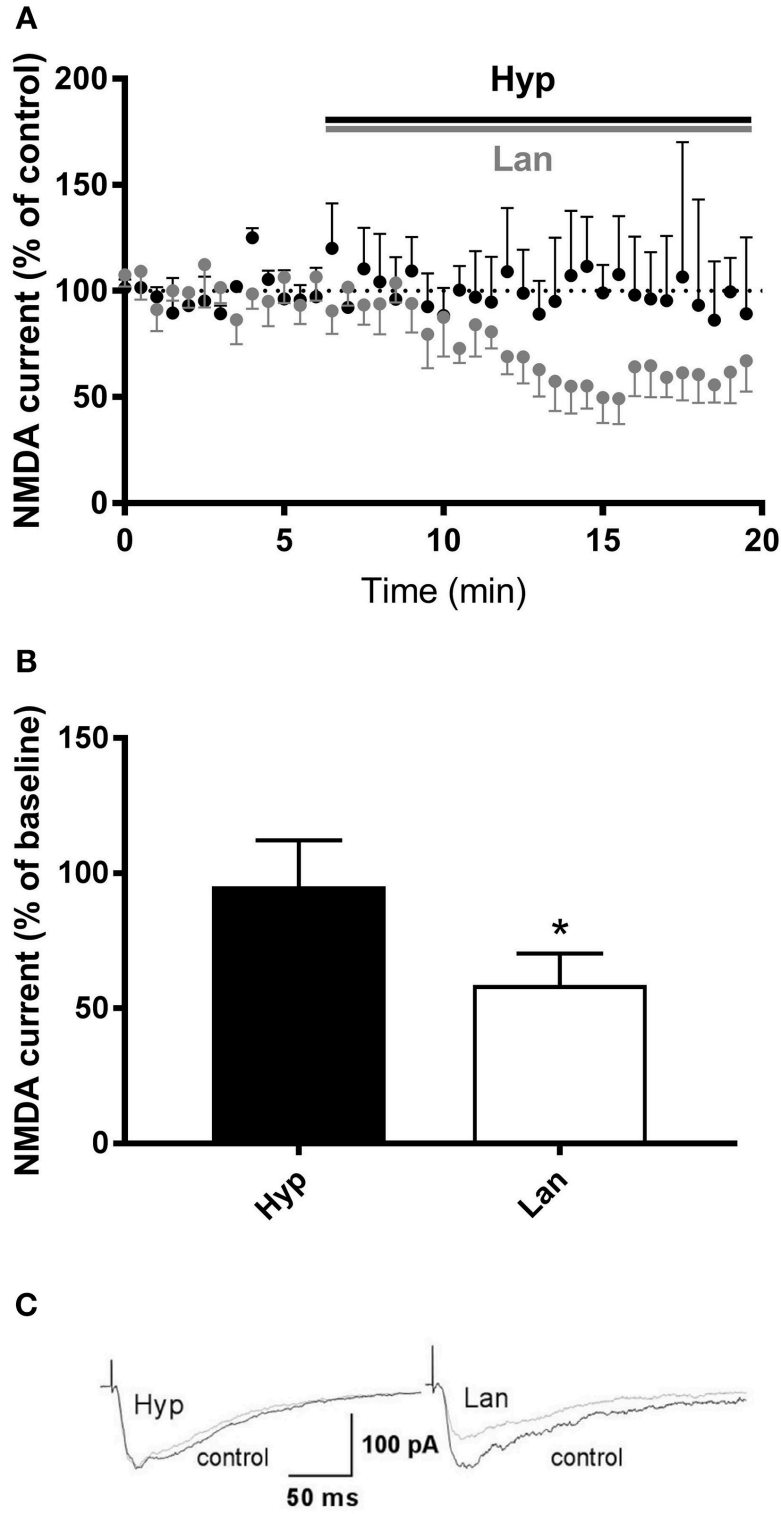
Effects of hyperforin/Lanicemine on layer V to later II/III NMDA synaptic transmission. **(A,B)** Application of Lanicemine markedly reduced the NMDA current, whereas hyperforin had no effect (*p* = 0.0313, median 51.63%, *n* = 6 vs. *p* = 0.8750, median 94.8, *n* = 4, respectively, Wilcoxon Signed Rank Test), *p < 0.05 vs. Hyp. Below, **(C)** Sample raw NMDA currents recorded under the two conditions, dotted line is for baseline currents.

Some studies have shown that hyperforin is able to influence NMDAR function (Chatterjee et al., 1999; Kumar et al., 2006). Thus, our next set of studies was focused on evaluating the effects of hyperforin and lanicemine on NMDAR-mediated synaptic currents resulting from electrical stimulation. As we described earlier, the behavioral effects of hyperforin were abolished by pretreatment with larixyl acetate and MK-2206 (TRPC6 and Akt 1/2/3 kinase inhibitors, respectively). Thus, for patch clamp studies, hyperforin's concentration (0.5 μM) was selected based on its potential to activate TRPC6 receptors (0.3 to 10 μM) (Tu et al., 2010; Heiser et al., 2013; Leuner et al., 2013). Electrophysiological studies showed that lanicemine (2 μM) significantly attenuated the NMDAR current while hyperforin had no effect (Figure 6). Moreover, there was no effect of acute hyperforin (1 μM) administration on NMDA receptor component amplitude (101% vs. 96%, *p* > 0.2) (Figure S2).

### The Affinity of Lanicemine and Hyperforin for NMDA Receptor Channel

The radioligand receptor binding studies (^3^H-MK801 as ligand) demonstrated no affinity for NMDA receptor channel of hyperforin In contrast lanicemine shows affinity for NMDAR receptors (K_i_ = 1.067 × 10^−5^) (Figure S3).

### The Effects of Hyperforin, Lanicemine, and Combined Administration of Hyperforin and Lanicemine on the Expression of Selected Proteins in the Frontal Cortex

**Figure 7:**
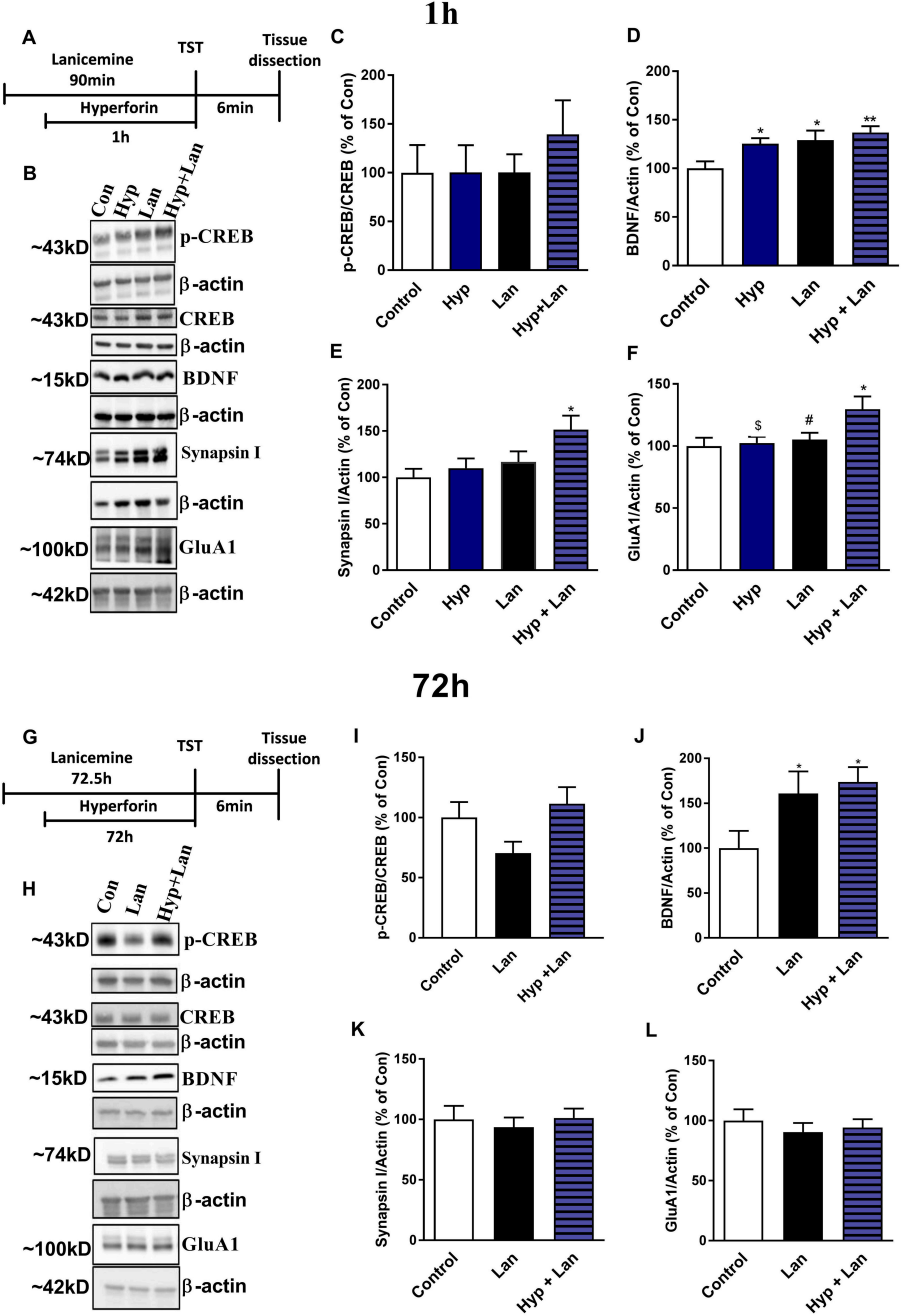
The effect of the administration of single doses of hyperforin (Hyp), lanicemine (Lan) and hyperforin + lanicemine (Hyp + Lan) on the expression of p-CREB, total CREB, BDNF, Synapsin I, and GluA1 subunit of AMPA receptors. **(A)** Experimental schedule of drug treatments. **(B)** Representative blots. **(C–F)** lanicemine (10 mg/kg) was administered 90 min and hyperforin (2.5 mg/kg) 60 min before TST. **(G)** Experimental schedule of drug treatments. **(H)** Representative blots. **(I–L)** lanicemine was administered 72.5 h and hyperforin 72 h before TST. Frontal cortex was dissected immediately after TST. **(C)** p-CREB/CREB [*F*_(3, 28)_ = 0.4795; *p* = 0.6992]. **(D)** BDNF, [*F*_(3, 28)_ = 4.886; *p* = 0.0074]. **(E)** Synapsin I, [*F*_(3, 24)_ = 3.573; *p* = 0.028]. **(F)** GluA1 [*F*_(3, 28)_ = 3.656, *p* = 0.0243]. *p < 0.05 vs. control, **p < 0.01 vs. control, ^#^*p* < 0.05 vs. Hyp + Lan, ^$^*p* < 0.05 vs. Hyp + Lan. Effects observed 72 h after treatment: **(I)** p-CREB/CREB, [*F*_(2, 20)_ = 2.734; *p* = 0.0892]. **(J)** BDNF [*F*_(2, 21)_ = 3.764; *p* = 0.0401]. **(K)** Synapsin I, [*F*_(2, 21)_ = 0.2062; *p* = 0.8153]. **(L)** GluA1, [*F*_(2, 21)_ = 0.3509; *p* = 0.7081]. *p < 0.05 vs. control. All data was analyzed by one-way ANOVA and Newman-Keuls multiple comparisons test. All values are expressed as mean ± S.E.M.

The antidepressant-like activity of NMDAR antagonists is related to the enhanced synthesis of synaptic proteins and neurotrophins (Duman et al., 2016). Hyperforin can activate signaling pathways involved in the processes of neuroplasticity *in vitro* (Heiser et al., 2013). Therefore, we determined the levels of the GluA1 subunit of AMPA receptors, synapsin I, BDNF, and CREB after treatment of mice with either hyperforin or lanicemine or a combination of both drugs (Figures 7A,B,G,H). This treatment strategy did not significantly alter p-CREB/CREB levels (Figure 7C) but elevated BDNF (Figure 7D) in the frontal cortex (1 h after hyperforin and 1.5 h after lanicemine administration). A single dose of either hyperforin or lanicemine did not alter the expression of synapsin I and GluA1 (Figures 7E,F). In contrast, the combined administration of hyperforin and lanicemine increased the levels of both proteins (Figures 7E,F). After 72 h, a combination of hyperforin and lanicemine did not alter p-CREB/CREB levels (Figure 7I); however, lanicemine and hyperforin + lanicemine significantly increased the levels of BDNF in the test animals compared to controls (Figure 7J). The levels of other proteins (synapsin I, GluA1) remained unchanged 72 h after treatment (Figures 7K,L).

### Lanicemine Potentiates Intracellular Ca^2+^ Responses Triggered by Hyperforin

**Figure 8:**
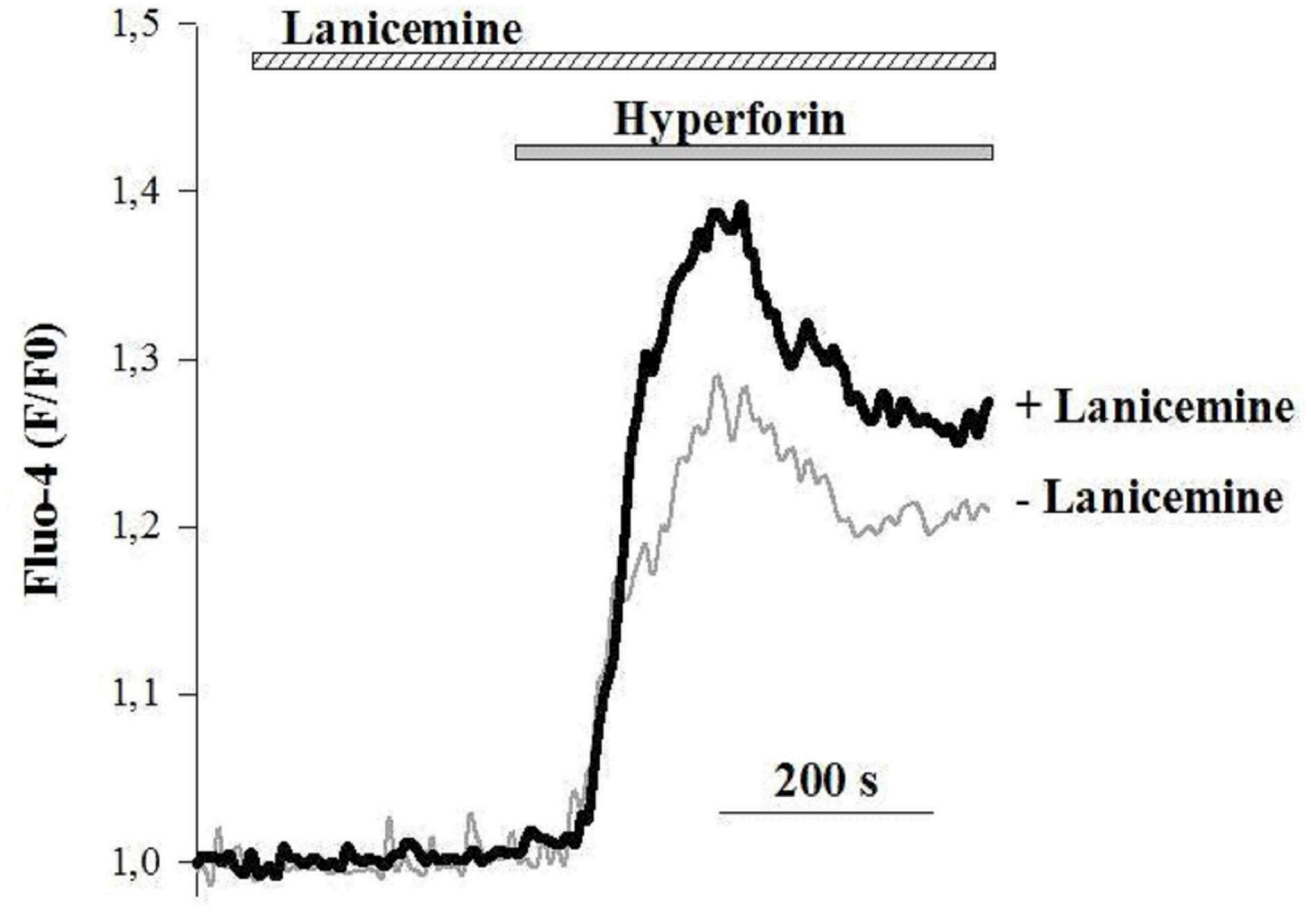
Representative Ca^2+^ responses from 2 different cells in response to the application of 1 μM hyperforin alone (gray line) and 4 min after the addition of 5 μM Lanicemine (black line). Note that Lanicemine remained during stimulation with hyperforin. *n* = 112 and 126 cells with hyperforin and hyperforin + Lanicemine, respectively. Hyperforin-induced Ca^2+^ responses were increased by 35 ± 6 % (*p* < 0.05, Student's *t*-test) by a 4 min pre-incubation with lanicemine.

To better delineate the effects of lanicemine, Ca^2+^ imaging experiments were carried out on primary cultures of cortical neurons loaded with the fluorescent Ca^2+^ probe Fluo4. The external application of hyperforin (1 μM) generated specific Ca^2+^ responses as illustrated in (Figure 8) (horizontal gray bar) (Chauvet et al., 2015, 2016). In some instances, lanicemine (5 μM) was added first (Figure 8, horizontal hatched bar). This had no effect on the basal Fluo-4 fluorescence, suggesting that basal levels of Ca^2+^ were not perturbed. However, hyperforin-induced Ca^2+^ responses were increased by a 4 min pre-incubation with lanicemine.

## Discussion

A rapid onset of action and long-lasting activity after a single dose of a drug are the most desired features sought for in novel antidepressants. These effects have been observed in rodents after treatment with ketamine and its metabolite, 2R,6R-hydroxynorketamine (Li et al., 2010, 2011; Autry et al., 2011; Zanos et al., 2016; Yang et al., 2017). A similar profile of antidepressant-like activity has also been described for a few NMDAR modulators (Ro 25-6981 and GLYX-13) (Li et al., 2010, 2011; Liu et al., 2017).

In the present study, we showed that a combination of a single dose of hyperforin and the NMDAR antagonist lanicemine evoked long-lasting antidepressant effects 72 h after administration in both naïve and chronic Cort-treated male mice. Additionally, in our preliminary study, we also observed the long-lasting antidepressant-like effects of hyperforin+lanicemine in naïve female mice. It seems that this interaction is not exclusive to lanicemine and hyperforin because the co-administration of hyperforin and MK-801, another NMDAR antagonist also induced the same long-lasting antidepressant-like responses in the TST in naïve mice. Lanicemine, MK-801, and hyperforin by themselves did not elicit long-lasting antidepressant effects after 72 h. It should be mentioned that we selected only a single dose each of lanicemine and MK-801; it is thus possible that the induction of the long-lasting antidepressant effects may be dose-dependent. As reported previously, the exceptional profiles of antidepressant-like activity of ketamine and NMDAR modulators are related to the immediate activation of molecular processes that lead to enhanced neuroplasticity mainly in the Hp and PFC (Li et al., 2010, 2011; Ardalan et al., 2017). These two brain structures are functionally impaired in MDD (Duman and Voleti, 2012; Duman et al., 2016). Fast and long-acting antidepressants enhanced the expression of synaptic markers like synapsin I or the GluA1 subunit of glutamate AMPA receptors (Li et al., 2010; Liu et al., 2017). In our study, hyperforin or lanicemine treatment did not alter the levels of synapsin I and GluA1 subunit in the frontal cortex of mice. However, a combined administration of the two compounds elevated the levels of synapsin I and GluA1 subunit after 1 h. Enhanced expression of GluA1 and synapsin I was not sustained 72 h after hyperforin + lanicemine treatment. These results may suggest that elevated synaptic protein synthesis is not involved in the long-lasting effects induced by hyperforin + lanicemine.

Another well-documented explanatory hypothesis of the mechanism of action of long-acting drugs is related to the enhanced activation of the BDNF signaling pathway. Results obtained from several studies have indicated that BDNF plays an important role in the mechanism of action of long-acting antidepressants. Autry et al. (2011) and Zanos et al. (2016) showed that a single dose of ketamine or its metabolite 2R,6R-hydroxynorketamine increased the synthesis of BDNF in the mouse Hp. Both ketamine and GLYX-13 have been shown to induce the release of BDNF in cortical neurons (Lepack et al., 2014, 2016). We found that hyperforin, lanicemine, and hyperforin + lanicemine enhanced BDNF expression 1 h after administration. Surprisingly, while only combined doses of lanicemine and hyperforin induced long-lasting antidepressant effects, lanicemine alone and lanicemine + hyperforin enhanced BDNF levels 72 h after administration. Because lanicemine similar to hyperforin + lanicemine also enhanced BDNF synthesis 72 h after treatment, these results may suggest that the enhanced synthesis of BDNF is not sufficient to explain the long-lasting antidepressant effects observed only after hyperforin+lanicemine treatment. As indicated in a few other studies, the relationship between BDNF synthesis and antidepressant-like activity may be more complicated than originally thought (Song et al., 2017). There are indications that BDNF may be synthesized in different parts of neural cells (glia vs. neurons or presynaptic vs. synaptic elements). These distinctions may underlie the biological role of BDNF in the neural system (Song et al., 2017). Therefore, it is possible that the significance of BDNF in the antidepressant-like activity of these compounds is not only related to its levels but also related to the site of synthesis. We should also mention that hyperforin is an agonist of TRPC6 receptors. Zhou et al. (2008) described the relationship between BDNF signaling pathway and TRPC6 receptors and showed that TRPC6 receptors mediated the influence of BDNF on spine formation in rat hippocampal cultures. The involvement of TRPC6 receptors in the antidepressant-like activity of hyperforin was implicitly revealed in our behavioral studies. We showed that pretreatment with larixyl acetate (TRPC6 antagonist) abolished the antidepressant-like effects induced by hyperforin in the TST. Also, pretreatment with MK-2206 (Akt1/2/3 kinase inhibitor) completely abolished hyperforin's effects observed in the TST. In other words, pharmacological blockade of TRPC6 and one of its main intracellular effectors Akt kinase inhibits the antidepressant effects of hyperforin induced in mice. These results are in agreement with *in vitro* studies which have shown that the biological effects of hyperforin are related to the activation of the TRPC6 channel (Leuner et al., 2007; Tu et al., 2010) followed by a subsequent activation of the Akt kinase (Heiser et al., 2013). Therefore, it is possible that the antidepressant-like activity of combined doses of hyperforin + lanicemine is dependent on crosstalk between the blockade of NMDAR, activation of TRPC6 receptors, and BDNF pathways. Additionally, (Zhou et al., 2008) showed that TRPC6 regulates synaptic plasticity through the CREB signaling pathway. It is also well-established that the synthesis of BDNF is dependent on the phosphorylation of CREB (Nibuya et al., 1996). So in the next step of our study, we evaluated the effects of treatments on p-CREB/CREB ratio levels. We only observed a trend toward an increase in the levels of p-CREB/CREB ratio 1 h after hyperforin + lanicemine treatment. Thus, the involvement of CREB in the biosynthesis of BDNF after hyperforin + lanicemine requires further detailed and precise studies.

Second possible hyperforin's antidepressant mechanism of action reported previously is related to blockade of NMDAR (Kumar et al., 2006). Whole cell patch clamp studies showed that hyperforin at 0.5 μM concentration did not change cortical NMDAR currents. Moreover, there was no effect of hyperforin (1 μM) administration on the NMDA component of the field potential. Furthermore, radioligand binding studies indicated that hyperforin had no affinity for the 3H-MK-801 labeled site of the NMDAR. Thus, in these particular conditions hyperforin does not interfere with NMDAR. Concomitantly, we have shown that pretreatment with NMDA abolished antidepressant-like activity of hyperforin in TST. To answer the question why NMDA administration blocks the antidepressant-like effect of hyperforin in TST in mice requires further investigation.

Because the biosynthesis and release of synaptic proteins like GluA1 and synapsin I, and BDNF and their release is strictly coupled to the activation of cellular processes that require elevated levels of intracellular Ca^2+^ (Duman et al., 2016; Finkbeiner, 2016) we sought to find out if the combined administration of hyperforin and lanicemine evoked different intracellular Ca^2+^ responses than the administration of either lanicemine or hyperforin alone. Calcium imaging studies revealed that lanicemine potentiated hyperforin-induced Ca^2+^ signals. However, the mechanism by which lanicemine regulates hyperforin-dependent Ca^2+^ responses is currently unknown and requires further studies.

To summarize, the results obtained in this study showed that treatment with a single active dose of hyperforin and lanicemine induced a long-lasting antidepressant-like activity in mice. These effects were observed in both naïve and corticosterone-treated male mice. Hyperforin + lanicemine effects were sustained for up to 6 days after treatment. Interestingly, our preliminary studies also showed that combined single doses of hyperforin + lanicemine also induced long-lasting effects in naïve female mice in the TST. The induction of antidepressant-like effects in both male and female mice is a very desirable feature of any potential, novel antidepressant because sex-related responses to antidepressant therapy have been postulated frequently (Khan et al., 2005). The potential benefit of administration hyperforin + lanicemine is associated with hyperforin's potential to improve cognitive activity. Because hyperforin attenuates cognitive disturbances induced by MK-801 it can be administered with other NMDAR antagonists which induce cognitive impairments. Furthermore, MDD with cognitive dysfunction has been described (Lam et al., 2014). Therefore, combined doses of hyperforin + lanicemine can be a useful potential strategy in the treatment of depression including cognitive disturbances. Another important question is what kinds of biological mechanisms are involved in hyperforin + lanicemine induced mechanism of action. Calcium imaging studies have shown that the biological interaction between hyperforin + lanicemine is a Ca^2+^-dependent process. Our biochemical and behavioral studies indicated that the main mechanism of action can be associated with crosstalk between the TRPC6 receptor and the BDNF signaling pathway. However, further studies are required to better understand the biological foundations of these interactions.

## Author Contributions

BP and GN designed the studies. BP, BS, AR-U, and KK performed behavioral studies. MS, JS, and KT conducted electrophysiological studies. AB performed calcium imaging studies. BP and BS performed surgical procedures. BP determined protein expression by Western Blotting. AS conducted radio ligand binding studies. BP, KF, and GN analyzed the results. BP, BS, and GN wrote the manuscript.

### Conflict of Interest Statement

The authors declare that the research was conducted in the absence of any commercial or financial relationships that could be construed as a potential conflict of interest.
