# Supplementary material for: Hyperforin Potentiates Antidepressant-Like Activity of Lanicemine in Mice
Source: Front Mol Neurosci. 2018 Dec 12;11:456. doi: 10.3389/fnmol.2018.00456 (PMC6299069; doi:10.3389/fnmol.2018.00456)
Supplement: Supplementary file 1 [file Data_Sheet_1.PDF]

Hyperforin potentiates antidepressant-like activity of lanicemine in mice

Supplemental Information

Frontiers in Molecular Neuroscience

Bartłomiej Pochwat[1], Bernadeta Szewczyk[1], Katarzyna Kotarska[1], Anna Rafało-Ulińska[1], Marcin Siwiec[2], Joanna E Sowa[2], Krzysztof Tokarski[2], Agata Siwek[3], Alexandre Bouron[4], Kristina Friedland[5], Gabriel Nowak[1,3]

[1]Institute of Pharmacology, Polish Academy of Sciences, Department of Neurobiology, Laboratory of Neurobiology of Trace Elements, Smętna Street 12, 31-343 Krakow, Poland

[2]Institute of Pharmacology, Polish Academy of Sciences, Department of Physiology, Smętna Street 12, 31-343 Krakow, Poland

[3]Faculty of Pharmacy, Jagiellonian University Medical College, Department of Pharmacobiology, Medyczna 9, 30-688, Krakow, Poland

[4]Université Grenoble Alpes, CNRS, CEA, BIG-LCBM, 38000 Grenoble

[5]Johannes Gutenberg University Mainz, Pharmacology and Toxicology, Insitute of Pharmacy and Biochemistry, Staudingerweg 5, 55128 Mainz, Germany

*Correspondence to: Bartłomiej Pochwat, PhD, Institute of Pharmacology, Polish Academy of Sciences, Department of Neurobiology, Laboratory of Trace Elements, Smętna Street 12, 31-343 Krakow, Poland

e-mail: pochwat@if-pan.krakow.pl

** Correspondence to: Gabriel Nowak, PhD, Institute of Pharmacology, Polish Academy of Sciences, Department of Neurobiology, Laboratory of Trace Elements, Smętna Street 12, 31-343 Krakow, Poland. 3Faculty of Pharmacy, Jagiellonian University Medical College, Department of Pharmacobiology, Medyczna 9, 30-688, Krakow, Poland

e-mail: nowak@if-pan.krakow.pl

Hyperforin potentiates the effects of lanicemine in the TST and FST in female mice

We examined the antidepressant-like activity of a combined administration of hyperforin (2.5 mg/kg) and lanicemine (10mg/kg). Lanicemine was administered 90 min before the TST and 30 min before hyperforin treatment (1A). FST was performed in the same mice 72h after hyperforin treatment (1B). Both at the 1h and 72h time point, combined treatment with hyperforin + lanicemine significantly reduced the immobility time (Figures 1A, B).

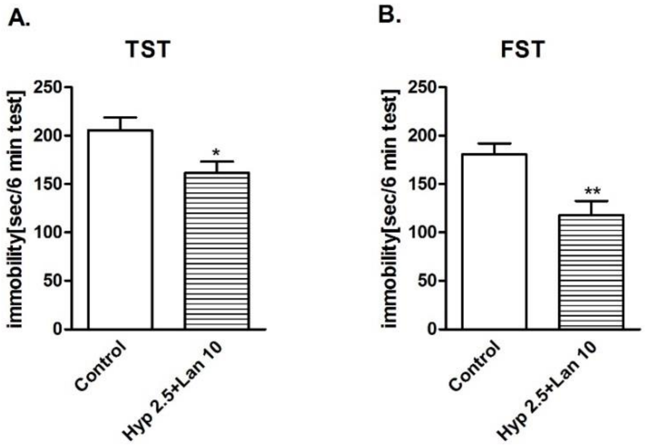

Figure S1.The effect of combined administration of a single dose of hyperforin (Hyp) and NMDAR antagonist: lanicemine (Lan) in TST (3a) and FST (3b) in female mice. Values are expressed as mean ± S.E.M (n=6) and analyzed using Student's t-test. *p<0.05; **p<0.01.

The effect of hyperforin administration on the NMDA component of the field potential

There was no effect of acute hyperforin (1 μM) administration on NMDA receptor component amplitude (101% vs 96%, p>0,2)

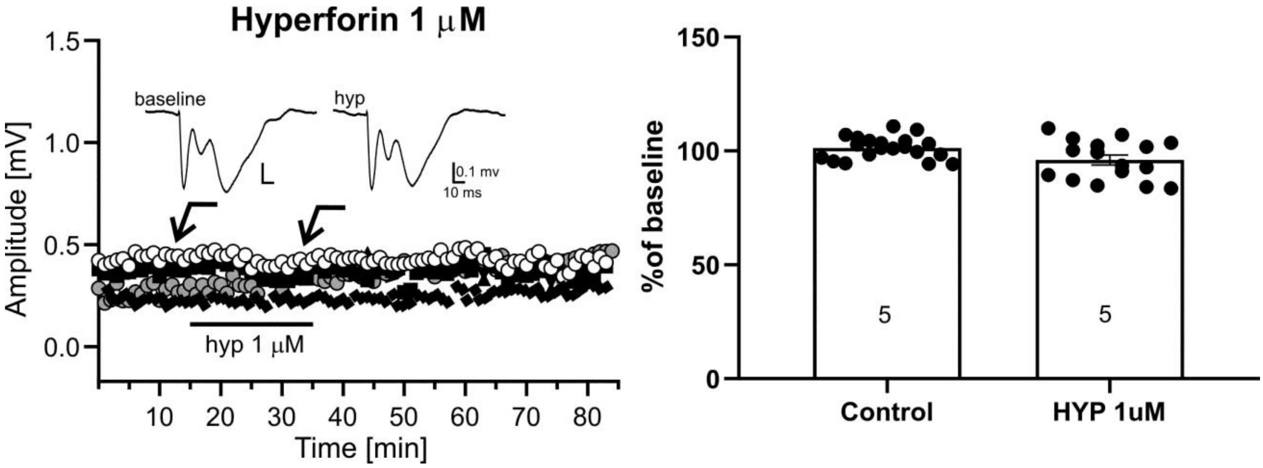

Figure S2. (A) Effect of 20min of Hyp administration (dark bar) on NMDA receptor-mediated component of field potentials. Insets: examples of the single field potentials recorded at times indicated by arrows, bar indicated amplitude (0.1mV) and length (10ms). (B) Mean (± SEM) amplitude of NMDA mediated component of FP recorded before and after Hyp administration. The numbers on the bars indicate the numbers of slices in each group. p > 0.2; Wilcoxon matched-pairs signed rank test.

The affinity of lanicemine and hyperforin for NMDA receptor channel

The radioligand receptor binding studies (^{3}H-MK801 as ligand) demonstrated no affinity for NMDA receptor channel of hyperforin In contrast lanicemine shows affinity for NMDAR receptors (K_i=1.067x10^{-5}).

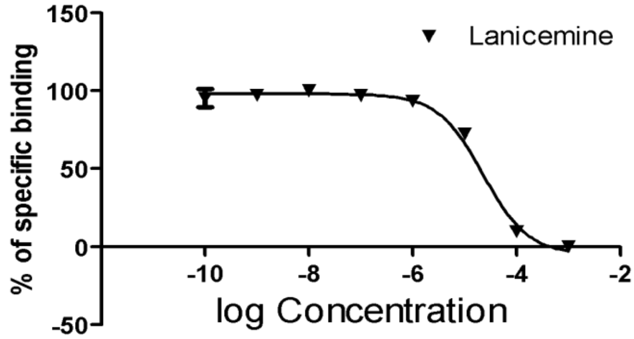

Figure S3. The effect of lanicemine on ^{3}H-MK801 radioligand binding.

Representative membranes from Western blot analysis

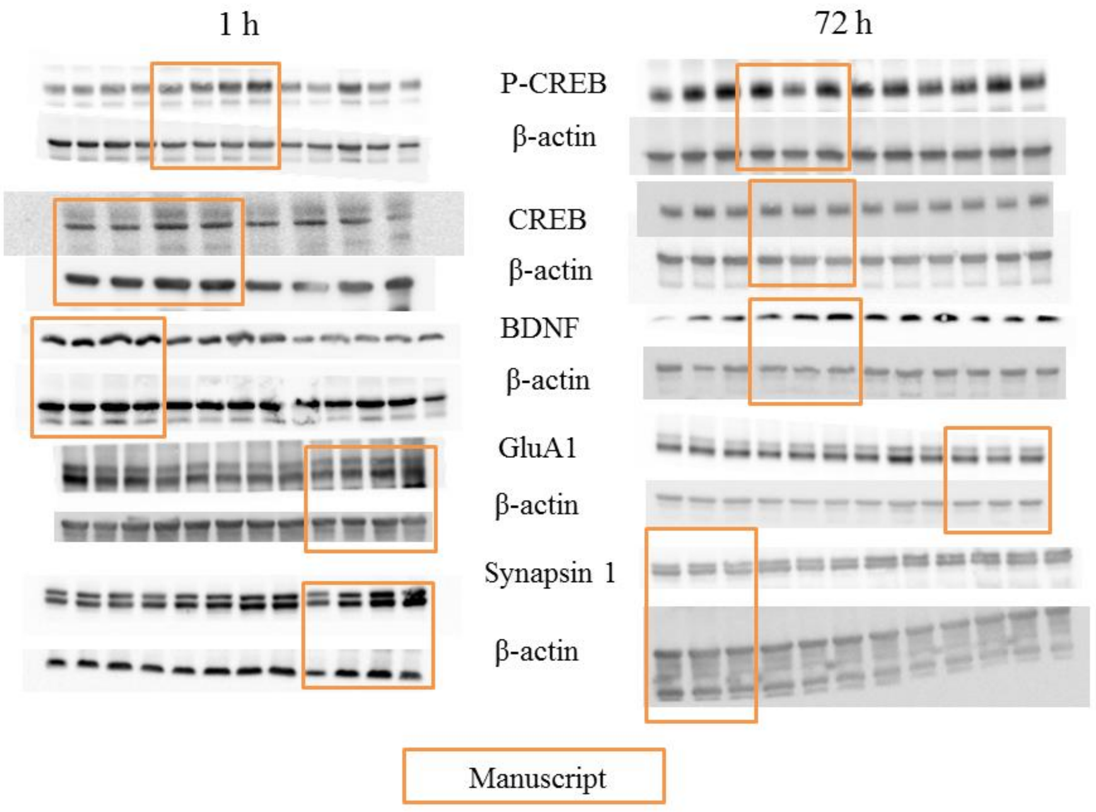

Figure S4. Images of western blot
